# 14-weeks combined exercise epigenetically modulated 118 genes of menopausal women with prediabetes

**DOI:** 10.3389/fendo.2022.895489

**Published:** 2022-08-15

**Authors:** Natália Yumi Noronha, Guilherme da Silva Rodrigues, Isabella Harumi Yonehara Noma, Camila Fernanda Cunha Brandao, Karine Pereira Rodrigues, Alexandre Colello Bruno, Chanachai Sae-Lee, Lígia Moriguchi Watanabe, Marcela Augusta de Souza Pinhel, Isabelle Mello Schineider, Mariana Luciano de Almeida, Fernando Barbosa Júnior, Déborah Araújo Morais, Wellington Tavares de Sousa Júnior, Torsten Plösch, Carlos Roberto Bueno Junior, Carla Barbosa Nonino

**Affiliations:** ^1^ Department of Internal Medicine, Ribeirão Preto Medical School, Ribeirão Preto, São Paulo, Brazil; ^2^ Department of Clinical and Toxicological Analyses, School of Pharmaceutical Sciences, University of São Paulo, São Paulo, Brazil; ^3^ Physical Education School, Minas Gerais State University, Divinópolis, Minas Gerais, Brazil; ^4^ Department of Radiotherapy, Ribeirão Preto Medical School Hospital and Clinics, University of São Paulo, Ribeirão Preto, São Paulo, Brazil; ^5^ Research Division, Faculty of Medicine, Siriraj Hospital, Mahidol University, Bangkok, Thailand; ^6^ Department of Health Sciences, Ribeirão Preto Medical School, Ribeirão Preto, São Paulo, Brazil; ^7^ Department of Molecular Biology, São José do Rio Preto Medical School, São José do Rio Preto, SP, Brazil; ^8^ College of Nursing of Ribeirão Preto, University of São Paulo, Ribeirão Preto, São Paulo, Brazil; ^9^ Department of Clinical Analysis, Toxicology and Food Sciences, School of Pharmaceutical Sciences of Ribeirão Preto, University of São Paulo, Ribeirão Preto, Brazil; ^10^ University Medical Center Groningen, Groningen, Netherlands; ^11^ Ribeirão Preto School of Physical Education and Sport, University of São Paulo, São Paulo, Brazil

**Keywords:** physical exercise, DNA methylation, combined physical exercise, epigenomics, older women

## Abstract

**Background:**

Pre-diabetes precedes Diabetes Mellitus (DM) disease and is a critical period for hyperglycemia treatment, especially for menopausal women, considering all metabolic alterations due to hormonal changes. Recently, the literature has demonstrated the role of physical exercise in epigenetic reprogramming to modulate the gene expression patterns of metabolic conditions, such as hyperglycemia, and prevent DM development. In the present study, we hypothesized that physical exercise training could modify the epigenetic patterns of women with poor glycemic control.

**Methods:**

48 post-menopause women aged 60.3 ± 4.5 years were divided according to their fasting blood glucose levels into two groups: Prediabetes Group, PG (n=24), and Normal Glucose Group, NGG (n=24). All participants performed 14 weeks of physical exercise three times a week. The Infinium Methylation EPIC BeadChip measured the participants’ Different Methylated Regions (DMRs).

**Results:**

Before the intervention, the PG group had 12 DMRs compared to NGG. After the intervention, five DMRs remained different. Interestingly, when comparing the PG group before and after training, 118 DMRs were found. The enrichment analysis revealed that the genes were related to different biological functions such as energy metabolism, cell differentiation, and tumor suppression.

**Conclusion:**

Physical exercise is a relevant alternative in treating hyperglycemia and preventing DM in post-menopause women with poor glycemic control.

## Introduction

Diabetes Mellitus (DM) is characterized by chronic hyperglycemia resulting from deficiencies in insulin secretion, insulin action, or both. Type 2 Diabetes (T2D) results from a progressive loss of pancreatic islet function followed by long-term insulin resistance and elevated insulin secretion (1) Prediabetes is an intermediate condition that precedes TD2. These conditions could occur during menopause and are related to sex hormone levels and aging changes. Postmenopausal women tend to have fluctuations in the hormone’s estrogen and progesterone, these changes affect how cells respond to insulin. The process of hormonal changes after menopause causes fluctuations in blood sugar levels in women (2). During menopause, women could also experience predictable symptoms, such as increased total cholesterol and triglycerides (TG), decreased high-density lipoprotein cholesterol (HDL-c), increased android fat distribution, and impaired vascular function (2). Preventive action to manage hyperglycemia should be appropriately implemented in the prediabetes stage since glycemic control is the common factor determining complications from DM, especially in menopause (2, 3).

In this context, studies have emphasized the importance of epigenetic regulation considering its role in activating or deactivating genes that impact metabolic outcomes (4). Studies using animal models demonstrated epigenetic variations associated with the susceptibility to the development of metabolic diseases, such as DM (5).

Another factor that could influence hyperglycemia and metabolic alterations is exposure to heavy metals in the environment. Some metals, such as lead (Pb^208^), can increase oxidative stress-inducing metabolic changes related to DM (6). Correlations between heavy metals and glycosylated hemoglobin (HbA1c), an indicator of hyperglycemia and long-term alterations in glucose metabolism, have been found in cross-sectional studies in France and Saudi Arabia (7).

Exercise training can promote health and improve metabolism through different mechanisms. The impact of exercise training on epigenetic programming has been generating exciting results (8). Yahaya (9) demonstrated that myocyte enhancer factor 2 (*MEF2)* migrates to the nucleus during exercise after being phosphorylated, interacting with peroxisome proliferative active receptor gamma coactivator 1A *(PPARγC1A)*, and histone acetyltransferases (HATs).

Physical exercise can increase the production of reactive oxygen species, both in skeletal muscle and in immune cells, leading to the development of oxidative stress, lipid, protein, and DNA modifications (10). Therefore, physical exercise can cause changes in DNA methylation (9, 11). Studies show that the response to physical exercise depends on genetic code and on the epigenetic signature, which can cause hypo or hypermethylation, of genes involved in muscle metabolism (9, 12). One study showed that chronic physical exercise caused hypomethylation of the promoter region of the nuclear receptor factor, fatty acid transporter (SLC27A4) and GLUT4 in patients with obesity and diabetes (13). Many studies involving physical exercise and DNA methylation, as Sailani et al. (14), showed that DNA methylation was lower in 714 promoter regions in physically active men compared to inactive men. Furthermore, it suggests that physical exercise is associated with the DNA methylation pattern increasing insulin sensitivity (14).

These modifications resulted in the acetylation of type 4 glucose transporter (*GLUT4)*, improving the expression of the gene and insulin tolerance in the muscles (15). Exercise training can also ameliorate the effects of heavy metal accumulation by increasing urinary elimination (16). Due to all these benefits, exercise training could prevent hyperglycemia and its complications. In the present study, we hypothesized that physical exercise training could modify the epigenetic patterns of women with poor glycemic control. Thus, we aimed to analyze if the 14-week combined physical exercise intervention could modify epigenetic variables and improve the glucose serum levels.

## Materials and methods

### Ethical aspects, study design, and participants

The present study was approved by the Ethics Committee for Research with Humans of the School of Physical Education and Sport of Ribeirão Preto, University of São Paulo (EEFERP-USP). The registration number is CAAE: 79582817.0.0000.5656. The study was also registered in the Brazilian Registry of Clinical Trials under RBR-3g38dx. Participants were recruited in the Physical Education Program for the Elderly at EEFERP – USP. The inclusion criteria were women between 50 and 70 years with a medical clearance certificate to perform physical exercise and who were sedentary at the baseline. The exclusion criteria included diagnostic limitations to physical training or physical test, current smokers, caffeine consumption in the last 24 hours before any blood draw, and more than 25% combined training absence.

Forty-eight post-menopause women participated in the study. They were divided into two groups according to the fasting blood glucose levels determined by the American Diabetes Society (2020): Pre-diabetes Group (PG), composed of 24 women with a fasting blood glucose level higher than 100mg/dL and the Normal Glucose Group (NGG), composed of 24 women with a fasting blood glucose level lower than 100mg/dL (9).

### Assessments

The anthropometric evaluation was assessed before and after 14 weeks of combined training. The height was measured using a vertical stadiometer - Balmak - EST-223 and weight using the G-Tech digital scale - Balgl200, G-Tech. Subsequently, the body mass index was calculated using the formula (weight/(height*height)) (17). Waist and hip circumferences were measured following the recommendations of Malachias et al. (17). Participants’ systolic and diastolic blood pressure was measured using an Automatic Digital Upper Arm Blood Pressure Monitor Meter (SBH, 2010 - OMRON^®^, model HEM-7113) (17).

The biochemical analyses included uric acid, total cholesterol, high-density lipoproteins (HDL-C), low-density lipoproteins cholesterol (LDL-C), triglycerides (TG), blood glucose, and insulin. We also calculated the HORMAR IR (Blood (mmol) x Insulin (Ul/mL) ÷ 22. 5).

To investigate the physical benefits of 14 weeks of combined training, the elbow flexion and extension, sitting and standing up from a chair, agility dynamics, and the six-minute walk test was adopted. Also, the 6MWT (m) methodology was used to measure aerobic capacity (18).

We also determined the total serum concentration of metals (Hg202, Al27, As75, Cd111, Pb208, Ni60, Li7) through inductively coupled plasma mass spectrometry (ICP-MS), following the protocol described by Batista et al. (19).

### Combined training

Combined training was performed for 14 weeks, three times a week, lasting 60 minutes. The training was divided into strength exercises (30 minutes) and aerobic training (treadmill or bicycle, with a total duration of 30 minutes) (14).

The training periodization followed the first two weeks. The participants performed two sets of 15 to 17 maximum repetitions and 50% of the aerobic training in the reserve heart rate. The participants started to do 10 to 12 maximum repetitions in the remaining weeks, and aerobic training became 70% of the reserve heart rate. The participants-maintained values ​​between three and six, a score presented on Borg’s subjective effort scale. The score we adopted on a scale from 0 to 10 represents moderate intensity. For the selection of exercises for strength training, the need to train the main muscles was considered, totaling five exercises for the muscles of the upper limbs and three exercises for the muscles of the lower limbs. Our group previously detailed the protocol (14).

### Large-scale DNA methylation array (Infinium-EPIC bead chips)

The DNA methylation was analyzed at the baseline and after the 14 weeks of training. Salting-out technique (20) was used to extract DNA from leukocytes. Biodrop spectrophotometry was used to assess DNA quality and concentration using. 1% agarose gel test was used to guarantee genomic DNA integrity. The run time was 90 minutes at 80W (21).

Following the manufacturer’s recommendations, the bisulfite method was applied using the DNA EZ methylation kit (Zymo Research, CA, USA). Illumina BeadChip EPIC Infinium Methylation BeadChip 850k was used for the methylation experiment. All recommendations for the Infinium^®^ HD Assay Methylation Protocol Guide were followed (22).

### Bioinformatics analysis

The raw data files were exported from iScan. A Samplesheet file was created to associate the collected information with raw data (23). The RStudio software (version 4.0.2) was used to process the methylation data. The data was organized as the participants’ codes, age, anthropometric, physical, biochemical, and metal information. The Sentrix Position column informs which row and column each sample was located in. The Sentrix_ID column provides the corresponding code of the BeadChip (23). The Bioconductor ChAMP data package was used for data normalization through the MyNorm function (24).

Differentially methylated regions (DMRs) were analyzed using the champ.DMR() function to compare PG vs. NGG groups. The bumphunter method was used to aggregate all probes into small groups. The random permutation method was applied to estimate DMRs with p < 0.05. Based on text mining and literature review, all DMRs were functionally annotated (24).

After identifying the DMRs inter and intra-group, the singular value decomposition (SVD) analysis method was used to evaluate the number and nature of significant components, correlating with various components of interest (24). Methylation data were normalized *via* minfi’s ssNoob (25). DNAmAge was calculated using the Methylclock package through the DNAmAge function (26). We also investigated the epigenetic clock of participants from both groups for intrinsic epigenetic age acceleration (IEAA) and extrinsic epigenetic age acceleration (EEAA). The epigenetic clock is a biomarker for age-related epigenetic changes as well as disease-specific changes (27). This clock has been recently developed by using specific DNA methylation sites to describe biological age at the level of the cell, tissue, and organ (28). The multi-tissue algorithm by Horvath generates age prediction, which highly correlates with chronological age above r=0.9 (29). In this study, DNAm Age was calculated using Horvath’s epigenetic clock algorithm using 353 CpGs (3).

In SVD analysis, the champ algorithm first does SVD deconvolution on the beta dataset. Then, the Random Theory Matrix method in the “isva” package is used to calculate the numbers of the latent variable and the top components of the SVD result. This analysis is comparable to a Principal Components Analysis (24)

### Statistical analysis

Two researchers verified the resulting data to maintain the values’ reliability and accuracy. The Statistica 7.0 software (StatSoft Statistica^®^) was used to perform statistical comparisons. The Shapiro-Wilk test tested the normality. The Levene test assessed the equality of variance between groups. Then, repeated-measures ANOVA was performed, considering p <0.05. Fisher’s *post hoc* compared all means and controlled the error rate at the significance level. Then, a Spearman correlation was performed for the non-parametric data and a Pearson correlation for the parametric data to identify possible correlations. The Student’s t-test compared means in the descriptive analysis.

## Results

### Effects of combined training in blood pressure, biochemical variables, and improvement of physical skills

The mean age of participants in the PG was 61.3 ± 5.2 years, while in the NGG was 59.9 ± 4.8 years (**Table 1**). The 14 weeks of combined training had an effect of time in systolic blood pressure (F=24.304; p<0.001), diastolic blood pressure (F=6.180; p=0.016), total cholesterol (F=7.191); p=0.010), HDL-C (F=8.168; p=0.006), TG (F=11.176; p=0.001), and glucose (F=25.281; p<0.001). No undesirable effects were noted, but we did not observe group-time effect or group-time interaction (**Table 1**).

**Table 1:** Mean ± standard deviation of anthropometric, blood pressure, and biochemical variables pre and post-14 weeks of combined training (PG: n = 24; NGG: n = 24).

Variables	Group	PRE	POST	p (group)	p (time)	p (group x time)
Age (years)	PG	61.3±5.2		0.184		
	NGG	59.9±4.8				
**Anthropometrics**						
Stature (m)	PG	1.6±0.1		0.199		
	NGG	1.6±0.1			
BM (kg)	PG	74.2±12.3	74.2±11.6	0.229	0.858	0.848
	NGG	70.3±8.2	70.2±8.0			
BMI (kg/m2)	PG	30.7±4.2	30.1±4.2	0.223	0.188	0.892
	NGG	28.2±3.2	27.8±2.8			
WC (cm)	PG	95.2±11.1	98.3±12.6	0.278	0.728	0.081
	NGG	94.1±10.3	92.2±9.4			
HC (cm)	PG	108.5±8.8	106.2±10.0	0.166	0.386	0.139
	NGG	103.6±7.3	104.2±6.2			
**Blood pressure**						
SBP (mmHg)	PG	140.9±20.1	126.7±15.1	<0.001^#^	<0.001^*^	0.192
	NGG	123.7±9.6	115.5±9.0			
DBP (mmHg)	PG	82.0±12.3	77.7±8.5	0.003^#^	0.016^*^	0.48
	NGG	74.0±6.7	71.6±5.4			
**Biochemistries**						
Uric acid (mg/dL)	PG	4.3±1.2	4.5±1.3	0.234	0.187	0.928
	NGG	4.0±1,1	4.1±1.0			
Cholesterol (mg/dL)	PG	220.2±37.0	207.2±38.1	0.614	0.010^*^	0.909
	NGG	212.8±33.1	201.9±29.1			
Glucose (mg/dL)	PG	115.1±24.0	104.9±12.0	<0.001^#^	0.23	0.05
	NGG	92.7±4.6	95.4±7.4			
HDL (mg/dL)	PG	50.7±9.4	48.9±8.9	0.026^#^	0.006^*^	0.114
	NGG	59.9±14.1	53.3±11.7			
LDL (mg/dL)	PG	135.6±37.1	131.1±37.3	0.299	0.198	0.848
	NGG	127.4±30.6	119.9±40.8			
Triglycerides (mg/dL)	PG	152.9±49.1	131.6±36.0	0.073	0.001^*^	0.654
	NGG	128.3±50.5	112.1±38.0			
Insulin UI/ml	PG	14.1±11.9	14.9±10.0	0.052	0.108	0.694
	NGG	8.8±4.9	10.5±5.1			
HOMAR IR	PG	71.5±63.8	71.7±54.3	0.018^#^	0.316	0.338
	NGG	36.4±19.6	44.7±22.7			

BMI, body mass index; WC, waist circumference; HC, hip circumference; SBP, systolic blood pressure; DBP, diastolic blood pressure; HDL, high-density lipoproteins LDL, low-density lipoproteins; CM, centimeters; mmHg, millimeters of mercury; kg, kilogram; m, meters; PG, prediabetes group; NGG, normal glucose group. *statistical difference time; ^#^statistical difference group; p < 0.05 for pre versus post (repeated measures two-way ANOVA was used).

We observed the effect of time on elbow flexion and extension (F=17.028; p<0.001) for upper limb strength; sitting and standing up from a chair (F=20.264; p<0.001) for lower limb; agility test (F=51.248; p<0.001); and six-minute walk test (F=23.872; p<0.001) for aerobic fitness. The effect of time in both groups demonstrated an improvement in physical skills after 14 weeks of combined training. No group-time effect or group-time interaction was observed (**Table 2**).

**Table 2:** Mean ± standard deviation of the PRE AND POST motor variables 14 weeks of combined training (PG: n 24; NGG: n 24).

**Variables**	**Group**	**PRE**	**POST**	**p (group)**	**p (time)**	**p (group x time)**
EFE (repetitions)	PG	18.5 ± 4.3	21.8 ± 4.4	0.705	<0.001^*^	0.929
	NGG	18.2 ± 3.7	21.3 ± 3.4			
SAS (repetitions)	PG	14.5 ± 4.5	18.4 ± 6.1	0.363	<0.001^*^	0.880
	NGG	13.7 ± 3.5	17.3 ± 4.2			
Agility (sec)	PG NGG	26.6 ± 3.2 25.9 ± 2.3	23.4 ± 3.6 21.7 ± 2.7	0.253	<0.001^*^	0.285
6MWT (m)	PG NGG	540.3 ± 67.1 532.3 ± 38.8	572.4 ± 60.3 584.2 ± 60.7	0.905	<0.001^*^	0.257

EFE, elbow flexion and extension; SAS, sit and stand; sec, seconds; m, meters; PG, prediabetes group; NGG, normal glucose group. *statistical difference time; p < 0.05 for pre versus post (repeated measures two-way ANOVA was used).

### Combined training decreases metals in both groups

**Figure 1:**
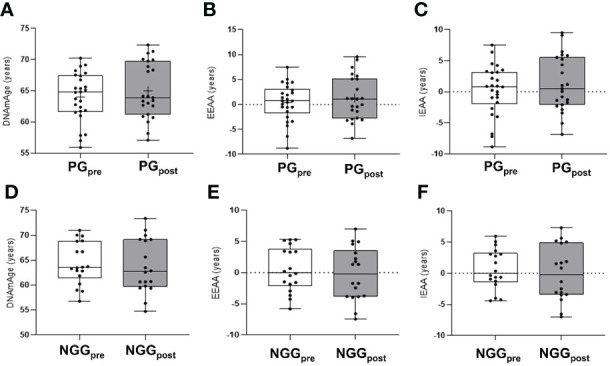
Differences in epigenetic age in PRE and POST comparisons 14 weeks of combined training. PG, prediabetes group; NGG, normal glucose group; EEAA, extrinsic epigenetic age acceleration; IEAA, intrinsic epigenetic age acceleration; Pre and pos-intervention comparison for the epigenetic clock in the PG group (panels **A–C**). Pre and post-intervention comparison for the epigenetic clock in the NGG group (panels **D–F**). (repeated measures two-way ANOVA was used). The presented values were not statically different between groups.

Analyzing metal levels and combined training together, we observed the effect of time decreasing mercury (Hg) (F=5.028; p=0.030) and lead (Pb) (F=12.100; p=0.001) in both groups. No effect or group-time interaction was observed (**Table 3**). There was a positive correlation between the arterial blood pressure and Pb^208^ in the PG_pre_ (PAS: r=0.389, p=0.003; PAD: r=0.337, p=0.010). After the intervention, the correlation was not significant. The epigenetic clock was not different between groups and did not change after the 14-week physical intervention (**Figure 1**).

**Table 3:** Mean ± standard deviation of PRE and POST metal values 14 weeks of combined training (PG: n 24; NGG: n 24).

**Variables (µg/L)**	**Group**	**PRE**	**POST**	**p (group)**	**p (time)**	**p (group x time)**
Hg	PG	0.06 ± 0.1	0.03 ± 0.1	0.651	0.030*	0.651
	NGG	0.05 ± 0.1	0.02 ± 0.1			
Al	PG	184.3 ± 150.7	159.5.1 ± 154.8	0.46	0.476	0.907
	NGG	152.2 ± 177.5	135.0 ± 59.0			
As	PG	2.9 ± 0.4	2.9 ± 0.2	0.386	0.818	0.181
	NGG	2.7 ± 0.2	3.0 ± 0.0			
Cd	PG	0.2 ± 0.8	0.03 ± 0.0	0.391	0.212	0.452
	NGG	0.8 ± 0.0	0.02 ± 0.0			
Pb	PG	2.1 ± 1.6	1.1 ± 0.3	0.385	0.001*	0.33
	NGG	1.7 ± 0.8	1.1 ± 0.0			
Ni	PG	6.3 ± 1.0	5.3 ± 1.3	0.175	0.44	0.879
	NGG	4.4 ± 2.4	3.0 ± 0.8			
Li	PG	0.6 ± 0.3	0.5 ± 0.1	0.492	0.104	0.934
	NGG	0.6 ± 0.3	0.5 ± 0.2			

Hg, mercury; Al, aluminum; As, arsenic; Cd, cadmium; Pb, lead; Ni, nickel; Li, lithium; PG, prediabetes group; NGG, normal glucose group. *statistical difference time; p < 0.05 for pre versus post (repeated measures two-way ANOVA.

### Differentially Methylated Regions after the 14 weeks of exercise compared with groups

Before the physical exercise training, we found 12 DMRs between the PG_pre_ and the NGG_pre_ (**Table 4**). After the 14 weeks of exercise, five regions remained Differentially Methylated between the two groups (**Table 5**).

**Table 4:** Differentially Methylated Regions (DMRs) of the Prediabetes Group (PG) compared to the Normal Glucose Group (NGG) before 14 weeks of physical activity intervention.

**NGG_pre_**	**PG_pre_**	**Gene**	***Δ β***	**p-value**
0.534	0.723	***IGR***	-0.19	<0.001
0.5	0.579	***RWDD3***	-0.08	0.013
0.25	0.317	***PM20D1***	-0.07	0.023
0.743	0.806	***WFIKKN2***	-0.06	0.015
0.747	0.784	***AURKC***	-0.04	0.026
0.199	0.231	***LOC441666***	-0.03	0.005
0.565	0.595	***HLA-DPB1***	-0.03	<0.001
0.104	0.131	***SLFN12***	-0.03	0.014
0.06	0.07	***CRYZ***	-0.01	0.007
0.045	0.035	***NPY***	0.01	0.004
0.059	0.049	***MIR596***	0.01	0.008
0.451	0.364	***DIP2C***	0.09	0.005

**Table 5:** Differentially Methylated Regions (DMRs) of the Prediabetes Group (PG) compared to the Normal Glucose Group (NGG) before 14 weeks of physical activity intervention.

**NGG_post_**	**PG_post_**	**Gene**	***Δ β***	**p-value**
0.699	0.495	***IGR***	-0.2	<0.001
0.259	0.188	***LOC441666***	-0.07	0.012
0.078	0.064	***CRYZ***	-0.01	0.004
0.592	0.585	***HLA-DPB1***	-0.01	0.001
0.036	0.048	***NPY***	0.01	<0.001

**Figure 2:**
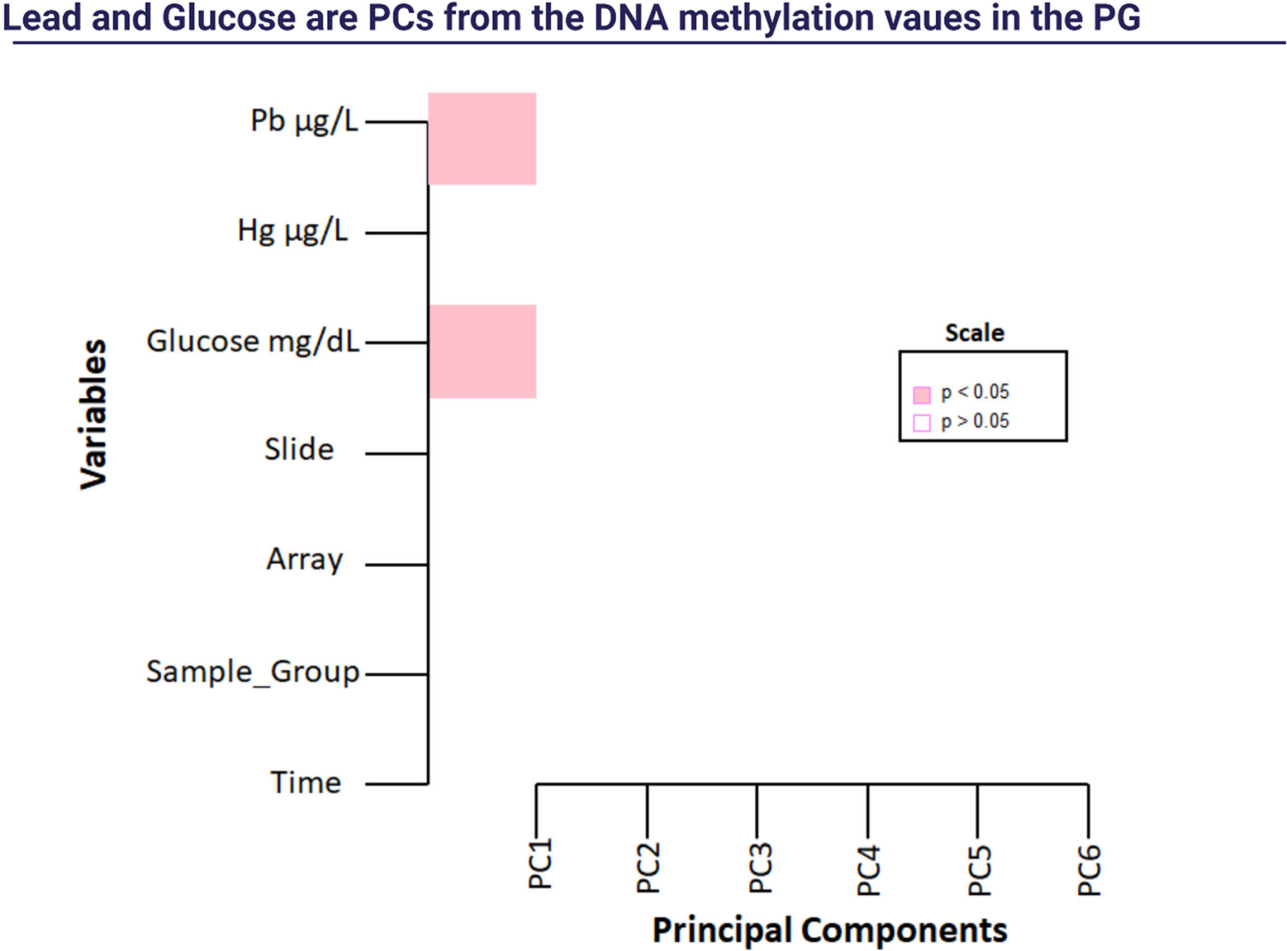
Singular Value Decomposition Analysis (SVD). Note: Pb: lead; Hg: mercury.

After the 14-week of exercise, we found 118 DMRs in the PG_post_ compared to the PG_pre_ (**Table 6**), and we performed a Singular Value Decomposition Analysis (SVD) to verify if the covariables of interest could explain the DNA methylation patterns (**Figure 2**).

**Table 6:** Differentially Methylated Regions (DMRs) from the PG_post_ compared to the PG_pre_ after 14-week physical intervention activity.

*Δ β* PG_post_ - PG_pre_			*Δ β* PG_post_ - PG_pre_		
	Gene	p-value		Gene	p-value
0.043	***CALD1***	0.004	-0.009	***HNRNPH1***	0.008
0.011	***MSI2***	0.024	-0.009	***ICK***	0.021
0.003	***RNF121***	0.024	-0.009	***AGL***	0.02
0	***SHFM1***	0.022	-0.009	***ANKRD11***	0.005
-0.001	***IDI1***	0.035	-0.009	***APH1A***	0.011
-0.001	***NUDT6***	0.024	-0.009	***ZSWIM7***	0.024
-0.001	***NDUFA4***	0.014	-0.009	**IGR**	0.02
-0.001	***APRT***	0.006	-0.009	***BCKDHA***	0.031
-0.001	***ATP6V1A***	0.01	-0.009	***NUP43***	0.028
-0.002	***WDR7***	0.01	-0.01	***RBPJ***	0.022
-0.002	***PLD3***	0.03	-0.01	***BDP1***	0.004
-0.003	***ZNHIT2***	0.02	-0.01	***COX8A***	0.011
-0.003	***ZNRD1***	0.019	-0.01	***GABPB2***	0.022
-0.003	***SLFN11***	0.013	-0.01	***TOM1L2***	0.026
-0.003	***FDPS***	0.008	-0.01	***PIGL***	0.016
-0.004	***FANCF***	0.005	-0.01	***HMGN4***	0.015
-0.004	***SNW1***	0.02	-0.01	***MOBKL3***	0.021
-0.004	***YIPF4***	0.008	-0.01	***HIST1H2BN***	0.004
-0.005	***ATP5A1***	0.012	-0.011	***CASP8AP2***	0.032
-0.005	***CHCHD8***	0.023	-0.011	***TAF15***	0.019
-0.005	***CEP97***	0.009	-0.011	***CENPK***	0.014
-0.005	***CCDC28A***	0.007	-0.012	***NECAP1***	0.007
-0.005	***PNPT1***	0.008	-0.012	***RANGAP1***	0.017
-0.005	***RBBP5***	0.011	-0.012	***EXOSC10***	0.013
-0.005	***NCRNA00188***	0.011	-0.012	***PSMB5***	0.009
-0.005	***GNAI3***	0.021	-0.012	***ZMYND11***	0.015
-0.005	***CWF19L1***	0.016	-0.013	***KAZALD1***	0.017
-0.006	***METTL3***	0.015	-0.013	***ERH***	0.023
-0.006	***ANAPC1***	0.028	-0.013	***FNTB***	0.007
-0.006	***LOC552889***	0.01	-0.013	***C7orf55***	0.024
-0.006	***NBR2/BRCA1***	0.01	-0.013	***GLIPR1L1***	0.008
-0.006	***CBY1***	0.004	-0.013	***GPATCH4***	0.012
-0.006	***C14orf101***	0.026	-0.013	***SRP14***	0.027
-0.007	***SLC4A1AP***	0.004	-0.014	***NRBF2***	0.022
-0.007	***AARS***	0.013	-0.014	***L3MBTL3***	0.027
-0.007	***C11orf65***	0.012	-0.014	***RND3***	0.032
-0.007	***DPY30***	0.025	-0.015	***ZNF684***	0.015
-0.007	***UBL7***	0.002	-0.015	***SNHG3/RCC1***	0.006
-0.007	**IGR**	0.015	-0.015	***MYOF***	0.002
-0.007	***BHLHE40***	0.029	-0.015	***TNFRSF10A***	0.017
-0.007	***COPZ1***	0.015	-0.015	***IDH3B***	0.024
-0.007	***H3F3B***	0.003	-0.015	***UFC1***	0.015
-0.007	***MCM2***	0.01	-0.017	***PCNP***	0.004
-0.007	***RPS27A***	0.003	-0.017	***MTMR12***	0.007
-0.008	**IGR**	0.007	-0.019	***RPL23A***	0.009
-0.008	***NCRNA00167***	0.005	-0.019	***C6orf52***	0.005
-0.008	***DBR1***	0.011	-0.019	***UFD1L***	0.011
-0.008	***C11orf60***	0.005	-0.02	***TNKS2***	0.011
-0.008	***SLC25A19***	0.025	-0.02	***ILF2***	0.004
-0.008	***SIDT1***	0.006	-0.02	***COX5B***	0.01
-0.008	***LEO1***	0.018	-0.02	***GNB2L1***	0.011
-0.009	***MED28***	0.004	-0.02	***C4orf14***	0.012
-0.009	***VWA5A***	0.018	-0.022	***WDTC1***	0.004
-0.009	***NR1H2***	0.027	-0.022	***HIST1H4C***	0.011
-0.009	***PES1***	0.028	-0.022	***PFDN2***	0.011
-0.009	***WDR62***	0.011	-0.024	***SNORD27***	0.01
-0.009	***MOCS3/DMP1***	0.009	-0.028	***CMTM6***	0.003
-0.009	***LSM5***	0.005	-0.033	***CNIH4***	0.008
-0.009	***COPS4***	0.02	-0.048	***KLF14***	0.018

**Figure 3:**
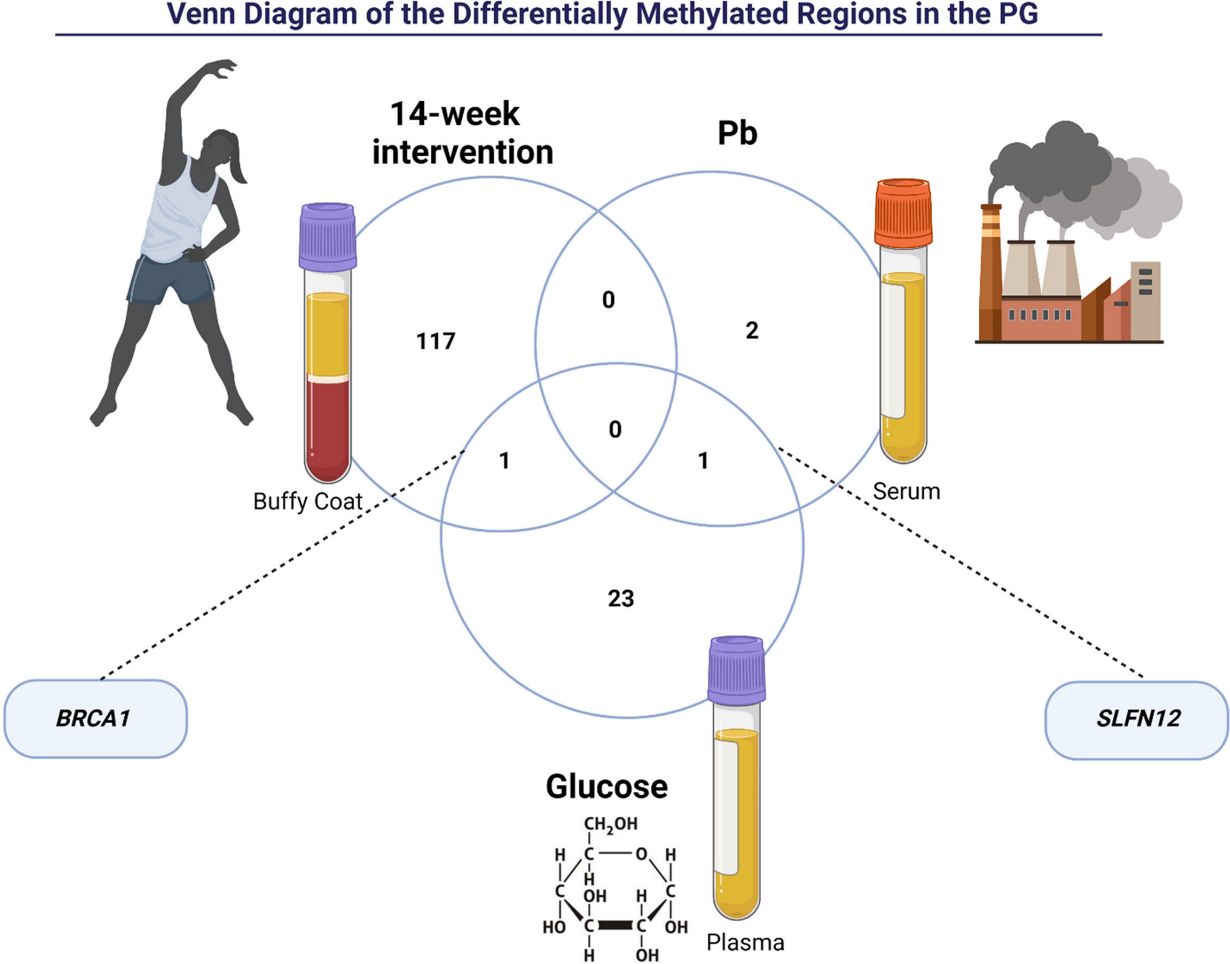
The intersection of Differently Methylated Regions of the prediabetes group (PG) between post-intervention, lead (Pb), and glucose. *BRCA1* gene is related to 14-week intervention and glucose. *SLFN12* gene is related to glucose and lead (Pb).

We also performed a Linear Regression of significant traits (Pb and glucose) and the DNA methylation targets of the PG group. We found three DMRs for Pb and 25 DMRs for glucose (**Figure 3**). *BRCA1* was hypomethylated after a 14-week physical exercise intervention and was associated with glucose in the PG group. There was also an interesting overlap between the DMRs related to Pb and glucose enriched in the gene *SLFN12*. Notably, this gene region was differently methylated in the PG_pre_ group than NGG_pre_ and associated with Pb in the PG. After the intervention, a decrease in Pb was observed, and the methylation patterns of *SLFN12* in PG_post_ became similar to NGG_post_.,,

## Discussion

### Combined training demonstrated benefits in physical and biochemical variables, providing positive results to cardiovascular health

One of the aims of this study was to investigate the effect of combined training on epigenetic patterns in the prediabetes (PG) and normal glucose group (NGG). Combined training is an alternative program involving aerobic and strength exercises in the same training session (30). Its effects are widely recognized in the literature for the numerous benefits to the cardiorespiratory system, prevention of oxidative stress, and critical metabolic alterations in the glycemic balance (31). According to Bellazi et al. (12), the regulating factor of blood glucose concentration is achieved due to the power of action of the three variables: insulin, adequate meals, and physical exercise (32). Indeed, after 14 weeks of training, we found a decrease in glycemic values in the PG group, and in both groups, we observed decreased levels of total cholesterol and TG levels, indicating an improvement in metabolic indicators of cardiovascular health.

Moreover, the combined training improved both groups’ systolic and diastolic blood pressure. A study by Schroeder et al. (13) observed that the combined training protocol was the only one that promoted significant changes in blood pressure values, with positive reductions in diastolic pressure (33). They demonstrated that the combined training had positive cumulative results in all cardiovascular outcomes (33).

Combined training also demonstrated benefits in functional capacity in our study, providing a time effect for the PG and NGG groups. These findings corroborated with Medeiros et al. (14), demonstrating an increase in maximal and functional strength after 12 weeks of training in 58 women aged 50-75 years (34).

Regarding heavy metal concentration, we observed a decrease in mercury and lead levels after 14 weeks of combined training. Another finding of our study was the positive correlation between lead and systolic and diastolic blood pressure values ​​in the PG_pre_ group. Lead is widespread in the environment, and even low blood levels of this toxic metal may raise the risk of cardiovascular diseases, stroke, and chronic kidney disease. It is estimated that in adult populations lead exposures account for 5% of the population attributable risk for high blood pressure. Further, single toxicant exposures provide some evidence for developmental nephrotoxicity demonstrated by altered estimated glomerular filtration rate (eGFR) and other kidney biomarkers, increasing a risk for nephropathy in individuals with T2D (35). In a cross-sectional study carried out by Asgary et al. (15), the authors demonstrated that lead, mercury, and cadmium levels were higher in patients with coronary artery disease (36).

### Differentially methylated regions in the prediabetes group are related to inflammation, insulin signaling, and cancer and are associated with a higher risk of T2DM

**Figure 4:**
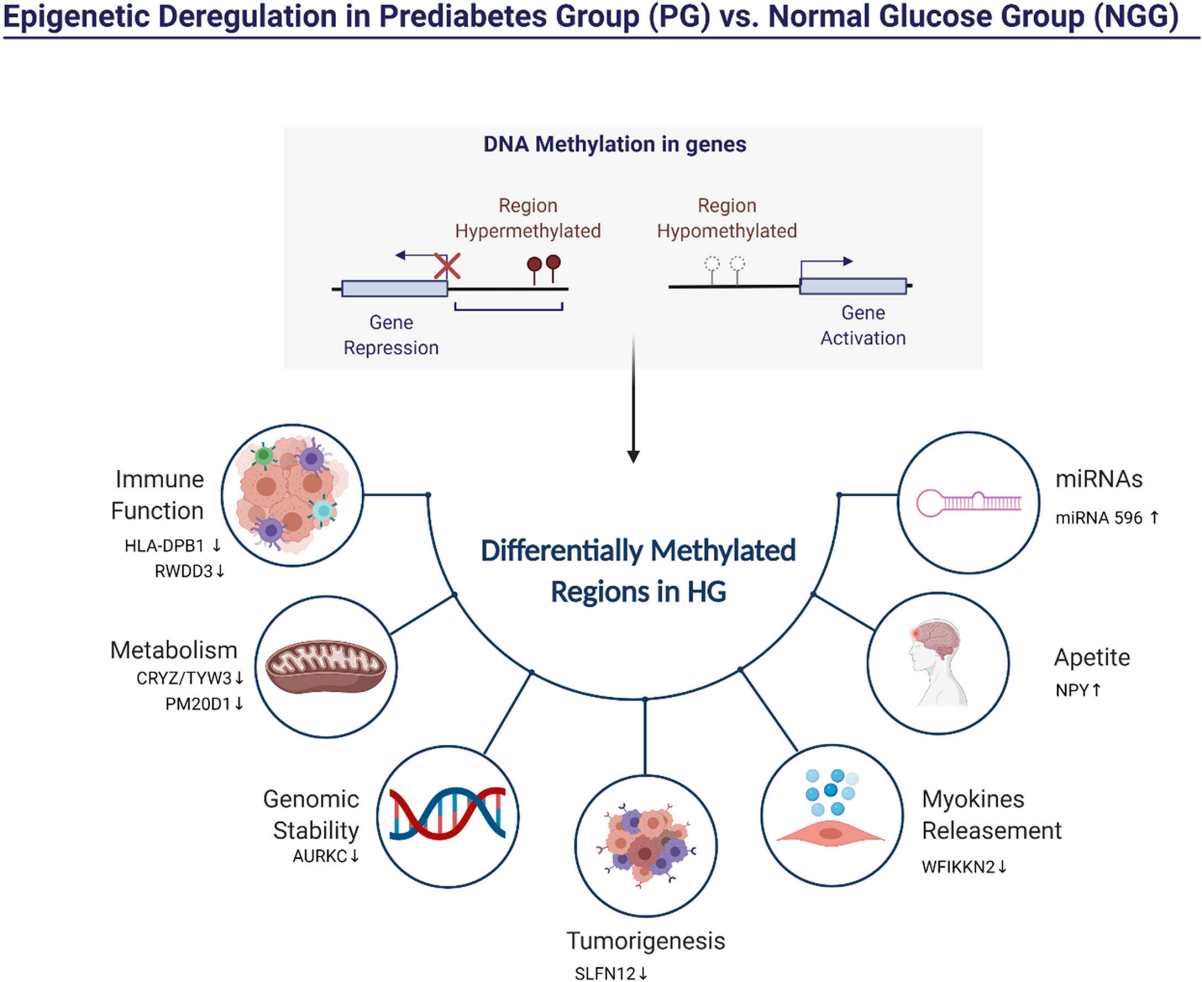
Representation of the pathways possibly influenced by differentially methylated regions (DMRs) in PG vs. NGG.

Our findings demonstrated that genes in differentially methylated regions of the PG group were related to inflammation and insulin signaling. Physical activity has improved glycemic control in adults with type 2 diabetes (27). Moreover, it can induce metabolic adaptations and changes in DNA methylation due to energy requirements (14). These changes in DNA methylation allowed for increased insulin sensitivity, expression of genes involved in energy metabolism, myogenesis, contractile properties, and oxidative stress (14). In the **Figure 4** is shown some metabolic pathways functionally enriched from the obtained DMRs.

**Figure 5:**
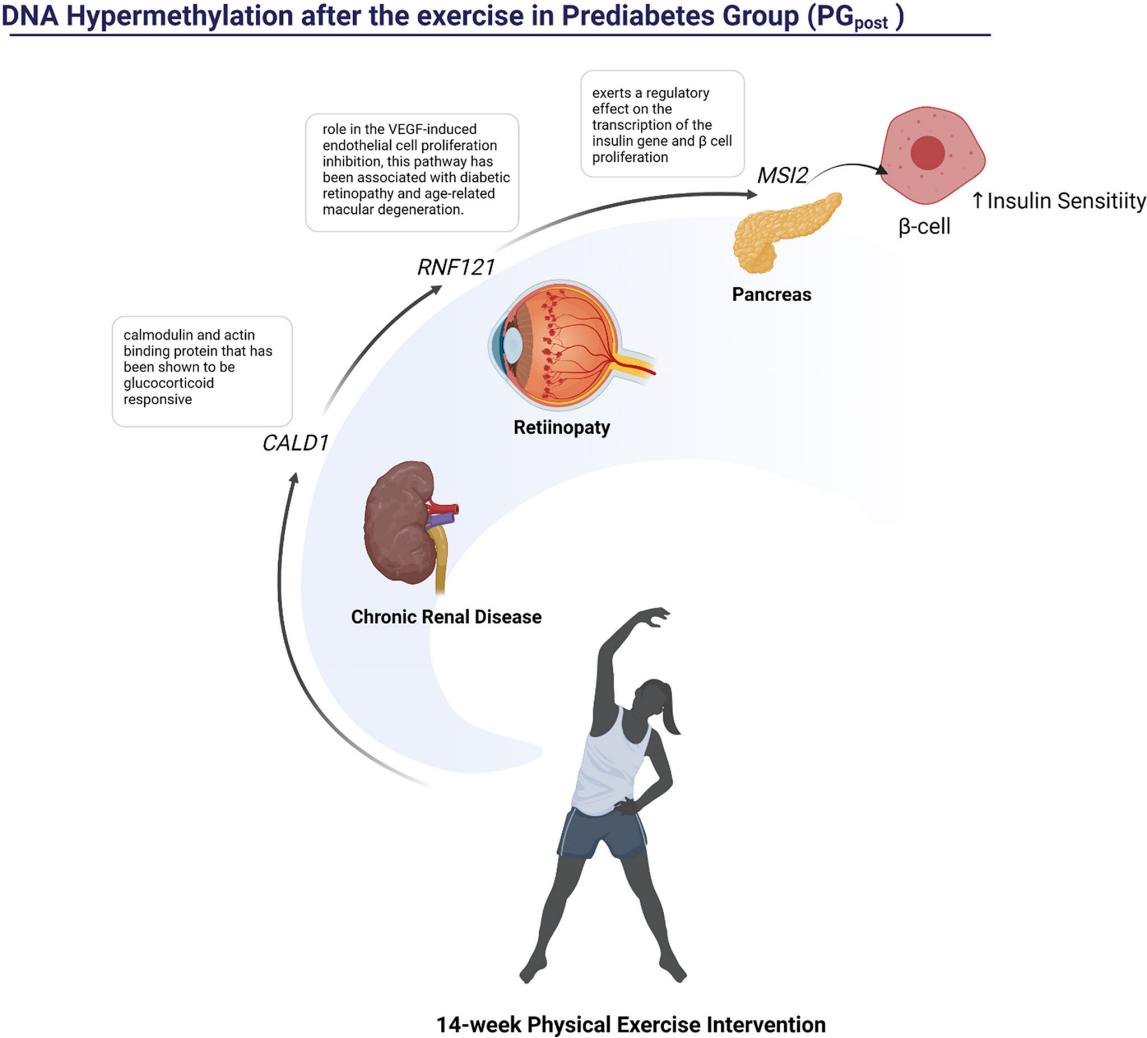
Representation of the pathways influenced possibly by hypermethylated regions in PG after the 14-week physical exercise intervention.

**Figure 5** shows hypermethylated pathways that are potentially altered after 14-week physical exercise intervention. Here we described specific hypermethylated genes’ role in PG observed in our study. *HLA-DPB1* plays a central role in the immune system by presenting peptides derived from extracellular proteins. Also, it is linked with inflammation and systemic insulin resistance (37). *RWDD3* gene also plays an immune function. The isoforms RWDD3 positively regulate the NF-kappa-β signaling pathway by enhancing the NF-kappa-β inhibitor, promoting its stabilization, and regulating the hypoxia-inducible factor-1 alpha (38). Other genes are found hypermethylated in PG group-related metabolism and genomic stability. The gene *TYW3/CRYZ* is related to levels of resistin. Previous studies reported associations between elevated circulating resistin levels and increased risk of cardiovascular disease (28). PM20D1 is an enzyme that condenses fatty acids and amino acids. The *PM20D1* locus shows genome-wide association studies (GWAS) with body mass index (BMI) and weaker associations with obesity-related conditions like type 2 diabetes and HDL-C levels (39). Aurora C kinases (AURKC) are essential kinases for cell division *via* regulating mitosis, especially the process of chromosomal segregation, associated with malignant cell transformation and genomic instability (16). PDE3A binding to SLFN12 results in cell killing and optimizing cancer therapeutics (40). WFIKKN2 is a follistatin domain-containing protein that binds GDF8/GDF11 proteins with high affinity, both of which have been implicated in DM development (41). The hypermethylation of these genes in individuals of the PG group could indicate a compromised metabolic function which could be associated with a higher risk of T2DM (42).

### The epigenetic reprogramming of genes after a 14-week physical exercise intervention can promote many alterations at a cellular level that regulates cellular metabolism in human metabolic diseases such as diabetes, obesity, and cancer

After the 14-week physical exercise intervention, 118 DMRs were detected. Three of these genes were hypermethylated: *CALD1*, *RNF121*, and *MSI2*. The *CALD1* was previously related to diabetes nephropathy (43), while the role of RNF121 in the VEGF‐induced endothelial cell proliferation inhibition has been associated with diabetic retinopathy and age-related macular degeneration (44). The *MSI2* hypomethylation has already been described as an epigenetic biomarker for hyperglycemic individuals by the Korean Genome Epidemiology Study (KoGES) (45).

**Figure 6:**
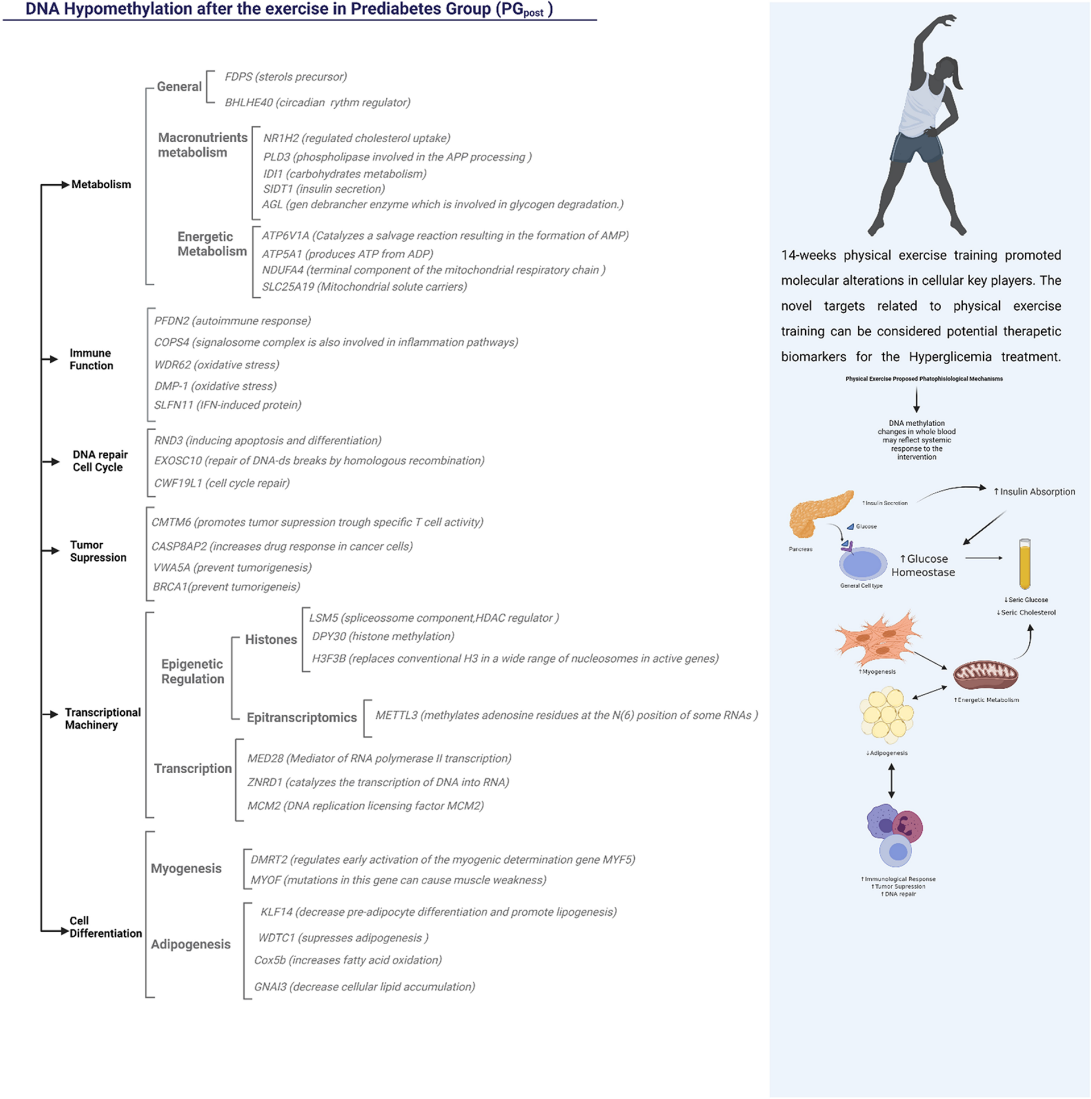
Representation of the functions possibly influenced by hypomethylated regions in PG after the 14-week physical exercise intervention. The Functional Annotation was done using the Genecards Database and literature text mining.

The additional 115 genes were hypomethylated and involved in many biological functions (**Figure 6**). Regarding the effects on general metabolism, the FDPS (*Farnesyl pyrophosphate synthase*) is a crucial enzyme for several classes of essential metabolites, including sterols, dolichols, carotenoids, and ubiquinones. The BHLHE40 Class E basic helix-loop-helix protein 40 is also a target that may influence the general metabolism due to its role as a transcriptional repressor in regulating the circadian rhythm and negatively regulating the activity of the clock genes and clock-controlled genes (46).

The mechanisms related to metabolism also included targets related to macronutrients, such as lipids and carbohydrates, which are important in the etiology of hyperglycemia (47). The NR1H2 (Oxysterols receptor LXR-beta; Nuclear receptor) gene was differentially methylated and can regulate cholesterol uptake through the ubiquitination of LDLR, VLDLR, and LRP8. Indeed, the PLD3 - Phospholipase D3 is an important key in lipids metabolism and may be involved in the Amyloid Precursor Protein (APP), already been related to Alzheimer´s Disease (AD). Three genes were differentially methylated in carbohydrate metabolism: IDI1, AGL, and SIDT1. ID1 has already been related to nephropathy, a condition connected to advanced stages of T2D (48). The AGL gene encodes the glycogen debrancher enzyme involved in glycogen degradation (49). The SIDT1 gene is associated with insulin secretion, and its expression was decreased in individuals with hyperglycemia (50).

Four differentially methylated genes were associated with energetic metabolism: ATP6V1A, ATP5A1, NDUFA4, and SLC25A19. The ATP6V1A is responsible for acidifying a variety of intracellular molecules, leading to HIF1A hydroxylation and catalyzing AMP formation (51). The ATP5A1 produces ATP from ADP (52). NDUFA4 is a cytochrome c oxidase subunit NDUFA4 and catalyzes oxygen reduction in water (53). The SLC25A19 mitochondrial thiamine pyrophosphate carrier is a transporter mediating thiamine pyrophosphate (ThPP) entrance into mitochondria (54).

The most interesting genes involved with the immune function were the COPS4 signalosome complex subunit 4, involved in phosphorylation of p53/TP53, c-jun/JUN, IkappaBalpha/NFKBIA, which leads to modulation of the inflammasome (55); theWDR62 and DMP-1 related to oxidative stress; and the SLFN11, interferon (IFN)-induced target (16).

The tumor suppression mechanisms, insulin resistance, and metabolic diseases have a common path (56). The CMTM6 depletion significantly decreases tumor-specific T cell activity (57). Indeed, the hypermethylation of two CpG sites upstream of the CASP8AP2 promoter influenced gene expression and treatment outcomes in lymphoblastic leukemia (58). In the present study, CASP8AP2 was hypomethylated after the intervention, suggesting that physical exercise training may decrease the risk of developing cancer in this population. The VWA5A plays a role in tumorigenesis as a tumor suppressor (59). BRCA1 is another important tumor suppressor, and studies have shown that individuals with hyperglycemia had this gene naturally more methylated than normoglycemic individuals (60).

Physical exercise can promote many alterations at a cellular level. Liu et al., 2020, demonstrated that miRNAs could epigenetically modulate the myogenesis and adipogenesis processes after a 12-week physical exercise intervention in older individuals (61). In the present study, important differentially methylated regions in the PG were related to myogenesis and adipogenesis after the intervention. The DMRT2 directly regulates early activation of the myogenic determination. It is required to initiate or maintain the proper organization of the sclerotome, dermomyotome, and myotome (by similarity). Mutations in the *MYOF* gene can cause muscle weakness, which is involved in membrane regeneration and repair. Reduced *KLF14* expression increases pre-adipocyte proliferation and decreases lipogenesis. In mice, the deletion of *KLF14* partially reproduces the human phenotype of insulin resistance, dyslipidemia, and T2D (62). The deregulation of the KLF14/PLK1 cascade plays a crucial role in thrombin-induced endothelial dysfunction in T2DM patients (63).

There is substantial epidemiological evidence that exposure to low levels of various environmental chemicals can influence the development of chronic metabolic diseases, including diabetes (64). It has been well demonstrated that lead shows potential genotoxicity to various research subjects under different conditions (65, 66). A study with human lymphoblastoid TK6 cells found that lead exposure results in DNA damage *via* promoting oxidative stress and the promoter methylation of DNA repair genes, such as *BRCA1* (67).

The *BRCA1* plays a critical role in DNA repair. Mutations in the gene that lead to loss of protein function can accumulate damaged DNA within the cell tumor-suppressor gene, which is strongly linked to an increased risk of breast cancer development (68). Recent studies demonstrated that BRCA1 is expressed in skeletal muscle and is a critical regulator of metabolic function in cultured human myotubes (69). Also, deletion of *BRCA1* expression in vascular cells results in the reduced catabolic breakdown of free fatty acids, and it can influence the metabolic phenotype of cultured cells and potentially the cells’ ability to respond to nutrient overload conditions (70).

Schlafens (*SLFN*) are intriguing proteins that play different roles in regulating cell proliferation, cell differentiation, immune cell growth and maturation, and inhibiting viral replication (17, 71).. However, it is still poorly understood how SLFNs interfere with the cell cycle machinery. It is a particularly tempting target for further research, as this gene is known to be down-regulated during T cell activation and up-regulated by type I interferons (IFNs) (18). It has also been suggested in cancer therapy since its expression was positively correlated with the growth inhibitory action of topoisomerase inhibitors on human cancer cells (19).

Acute exercise seems to be associated with active demethylation pathways. The mechanism proposed for active demethylation involves ten-eleven translocation (TET) proteins, suggesting that metabolites produced during exercise could alter the activity of TET proteins (20).

This study’s strengths were demonstrating the modulation of biochemical and physical parameters after a physical exercise intervention between different groups. In addition to verifying differently methylated regions in the PG group compared to the NGG group using a large-scale DNA methylation array (Infinium-EPIC beadchips) and the effect of combined training. The limitations were that analyzes of RNA expressions of the differentially methylated genes were not performed.

## Conclusions

The 14-week combined physical exercise intervention led to the modulation of biochemical and physical parameters and the epigenetic reprogramming of genes involved in many biological functions such as metabolism, cell differentiation, tumor suppression, and immune function. Moreover, exercise training decreases mercury and lead serum concentrations. In future studies, longer intervention could be proposed to check the sustained effects of long-term combined physical exercise practice.

## Data availability statement

The datasets presented in this study can be found in online repositories. The names of the repository/repositories and accession number can be found below: GEO, GSE199700.

## Ethics statement

The present study was approved by the ethics committee for Research with Human of the School of Physical Education and Sport of Ribeirão Preto, University of São Paulo (EEFERP-USP). The registration number is CAAE: 79582817.0.0000.5656. The study was also registered in the Brazilian Registry of Clinical Trials under RBR-3g38dx. The patients/participants provided their written informed consent to participate in this study.

## Author contributions

Conceptualization: NN and GR; methodology: NN, IN, and GR; formal analysis: NN, GR, CS-L, AC, and KR; resources: CBu and CN; data curation: GR, NN, and IN; performed experiments: NN, GR, LW, MP, DM, WS, and MA; writing—original draft preparation: NN, GR, IN, TP, CBr, and KR; writing—review and editing: TP, NN, GR, IN, and CBr; visualization: All authors; supervision: FB, CBu, and CN; funding acquisition: FB, CBu, and CN. All authors contributed to the article and approved the submitted version.

## Funding

São Paulo Research Foundation (FAPESP) (#2017/21361-2) and (#2018/24069-3); National Council for Scientific and Technological Development (CNPq: #408292/2018-0). Personal funding: (FAPESP: #2014/16740-6; #2020/08687-9) and Academic Excellence Program from Coordination for higher Education Staff Development (CAPES:88882.180020/2018-01) and (CAPES:88882.180033/2018-01).

## Acknowledgments

We would like to thank the Graduate Program in Clinical Medicine at the University of São Paulo, Ribeirão Preto, Foundation for Support of Teaching, Research and Assistance at HCFMRP-USP, Faculty of Medicine of São José do Rio Preto and School of Physical Education and Sport at the University from Sao Paulo and FAPEMIG (Foundation for Research Support of the State of Minas Gerais), process APQ-02169-21.

## Conflict of interest

The authors declare that the research was conducted in the absence of any commercial or financial relationships that could be construed as a potential conflict of interest.

## Publisher’s note

All claims expressed in this article are solely those of the authors and do not necessarily represent those of their affiliated organizations, or those of the publisher, the editors and the reviewers. Any product that may be evaluated in this article, or claim that may be made by its manufacturer, is not guaranteed or endorsed by the publisher.
